# Whole transcriptome analysis and gene deletion to understand the chloramphenicol resistance mechanism and develop a screening method for homologous recombination in *Myxococcus xanthus*

**DOI:** 10.1186/s12934-019-1172-3

**Published:** 2019-07-10

**Authors:** Ying-jie Yang, Raghvendra Pratap Singh, Xin Lan, Cheng-sheng Zhang, Duo-hong Sheng, Yi-qiang Li

**Affiliations:** 1 Marine Agriculture Research Center, Tobacco Research Institute, Chinese Academy of Agricultural Sciences, Qingdao, 266101 Shandong China; 2 State Key Laboratory of Microbial Technology, Shandong University, Qingdao, 266237 Shandong China; 3 Department of Research & Development, Biotechnology, Uttaranchal University, Dehradun, 248007 India; 4 Department of Bio-chemistry, Qingdao Technical College, Qingdao, 266555 People’s Republic of China

**Keywords:** Transcriptome, *Myxococcus xanthus*, Chloramphenicol resistance, Antibiotic selection system

## Abstract

**Background:**

*Myxococcus xanthus* DK1622 is a model system for studying multicellular development, predation, cellular differentiation, and evolution. Furthermore, it is a rich source of novel secondary metabolites and is widely used as heterologous expression host of exogenous biosynthetic gene clusters. For decades, genetic modification of *M. xanthus* DK1622 has mainly relied on kanamycin and tetracycline selection systems.

**Results:**

Here, we introduce an alternative selection system based on chloramphenicol (Cm) to broaden the spectrum of available molecular tools. A chloramphenicol-resistant growth phase and a chloramphenicol-susceptible growth phase before and after chloramphenicol-induction were prepared, and later sequenced to identify specific genes related to chloramphenicol-repercussion and drug-resistance. A total of 481 differentially expressed genes were revealed in chloramphenicol-resistant Cm5_36h and 1920 differentially expressed genes in chloramphenicol-dormant Cm_8h. Moreover, the gene expression profile in the chloramphenicol-dormant strain Cm_8h was quite different from that of Cm5_36 which had completely adapted to Cm, and 1513 differentially expression genes were identified between these two phenotypes. Besides upregulated acetyltransferases, several transporter encoding genes, including ABC transporters, major facilitator superfamily transporters (MFS), resistance-nodulation-cell division (RND) super family transporters and multidrug and toxic compound extrusion family transporters (MATE) were found to be involved in Cm resistance. After the knockout of the most highly upregulated MXAN_2566 MFS family gene, mutant strain DK-2566 was proved to be sensitive to Cm by measuring the growth curve in the Cm-added condition. A plasmid with a Cm resistance marker was constructed and integrated into chromosomes via homologous recombination and Cm screening. The integration efficiency was about 20% at different concentrations of Cm.

**Conclusions:**

This study provides a new antibiotic-based selection system, and will help to understand antibiotic resistance mechanisms in *M. xanthus* DK1622.

**Electronic supplementary material:**

The online version of this article (10.1186/s12934-019-1172-3) contains supplementary material, which is available to authorized users.

## Background

The gliding Gram-negative Myxobacteria, belonging to the delta division of Proteobacteria, are well known for social motility in space and time. They are known to swarm, predate, have multicellular differentiation, grow millimeter-size spore-filled fruiting bodies, and have large genomes of more than 9 Mb in size [1–4]. The genus *Myxococcus*, especially *M. xanthus* DK1622 represents as a model strain among the myxobacteria since its life-cycle can be investigated under laboratory conditions. Furthermore, *M. xanthus* DK1622 can be used as a heterologous expression host to express secondary metabolite gene clusters from other difficult-to-handle myxobacteria or marine myxobacteria for the production of metabolites, e.g. epothilone, haliangicin, disorazol, etc. [5–8]., which can be scaled up by genetic modification of the genome. Currently, however, operation of the *M. xanthus* DK1622 genome in the laboratory is governed mainly by antibiotic resistance against kanamycin and tetracycline [9, 10].

Chloramphenicol (Cm) is a broad-spectrum antibiotic, characterized by the presence of a nitrobenzene group containing nonionic chlorine, an amide linkage involving a derivative of dichloroacetic acid and a propanol moiety [11]. It binds directly to the 50S ribosomal subunit and inhibits peptide bond formation by interacting with the peptidyl transferase center [12]. There are three main mechanisms responsible for resistance to Cm:Cm acetyltransferases (CAT) encoded by *cat* genes [11], Cm efflux pumps mediated by numerous kinds of transporters [13–16] and rRNA methylase [17]. Some *cat* genes are inducible and expressed via translational attenuation, with Cm itself acting as an inducer [18]. A Cm-resistant phenotype of *M. xanthus* FBt was derived and characterized from *M. xanthus* FB, in which Cm acetyltransferase activity was shown to be related to Cm resistance [19]. The *cat* gene belonging to Cm acetyltransferase is often used as a Cm resistance marker in many organisms, as a genetic operation tool [8, 20].

Here, we compared the transcriptome of wild-type phenotype DK1622 with that of other phenotypes under the influence of Cm, to understand the mechanism by which DK1622 grows normally in the presence of Cm, and to develop an antibiotic selection tool. Here, a total of 297 genes were identified to be highly increased in our Cm resistant phenotype (Cm5_36h). Gene knockouts and comparative growth analysis indicated that MXAN_2566 was the main player involved in Cm resistance; a gene encoding a protein that pumped Cm outside the cell. In gene-knockout strain DK-2566, a method to integrate gene fragments into the genome via homologous recombination using Cm selection, with our constructed Cm marker plasmid was developed successfully. It add to a growing toolkit for molecular studies in *M. xanthus* and will be useful for the elucidation of patterns in microbial cell factories.

## Results

### *M. xanthus* DK1622 grows normally in CTT + Cm medium after induction

**Fig. 1:**
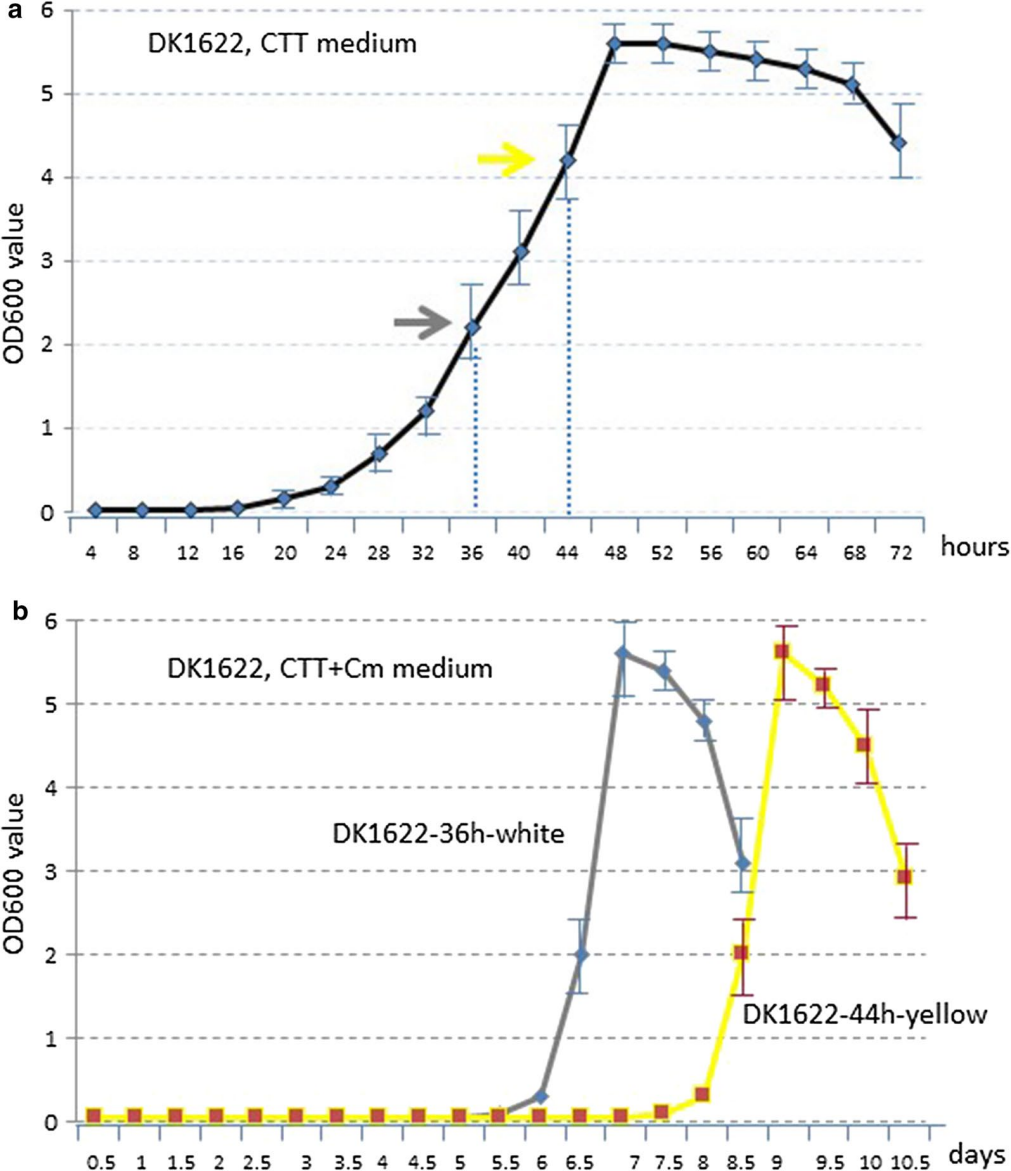
Growth curves of *M. xanthus* DK1622 in CTT liquid medium with or without Cm addition. **a** Growth curves of *M. xanthus* DK1622 in CTT liquid medium without Cm addition. **b** Sensitivity of *M. xanthus* DK1622 cultivated from different growth stage with Cm addition (10 μg/ml). Cells from exponentially growing culture in the absence of Cm, at different time points where cells stay white or become yellow, were diluted to OD 600 of 0.05 in CTT medium, marked in CTT-white or CTT-yellow. Cell density was monitored after incubation at 30 °C for 12 h. Experiments were performed in triplicate. Error bars indicate standard deviations

Previously, the *cat* gene was introduced into the genome of *M. xanthus* via either transposition or site-directed insertion (Mx8) [8]. Subsequently, the *cat* gene was used as a marker, flanked by two loxP sites, to indicate the cut efficiency by Cre recombinase in *M. xanthus* DK1622 [21] in our lab (which was successful). When we tried to integrate homologous arm into chromosome by Cm screening, we could not obtain the correct mutant strains from Cm resistant strains. Hence, we studied Cm-induced growth of DK1622 in order to construct a sensitive strain for chassis construction and the development of genetic operation tools. To determine the effect of Cm on growth, DK1622 cells at the mid-exponential phase and later-exponential phase were used as seed broths, and diluted to fresh CTT liquid medium supplemented with Cm. At these two time points, cells were white (36 h of incubation; OD 600 was about 2.3) and were observed to become yellow later (44 h of incubation; OD 600 was about 4.1). These cells were marked as DK1622-36 white or DK1622-44h yellow (Fig. 1b), respectively.

Differently from the DK1622 wild type, which only required 48 h to reach the stationary phase at 30 °C in CTT liquid medium (Fig. 1a), DK1622-36 white in CTT + Cm liquid medium required 6-days to enter the exponential phase through a dormant stage. DK1622-44 yellow (in CTT + Cm liquid medium) had spent 8-days in dormancy, which is longer than DK1622-36 white. During the log phase, cells remained yellow (Fig. 1b). In the subsequent experiment, DK1622-36 white was used with CTT + Cm liquid medium to obtain the Cm5_36h phenotype.

To investigate whether the Cm5_36h phenotype can grow normally in CTT + Cm, we measured the generation time of phenotype Cm5_36h. Cm5_36h of *M. xanthus* grew with a mean generation time of 5.3 h. In the absence of Cm, the mean generation time of strain DK1622 was 5.1 h. Hence, there was no significant difference in the mean generation time of Cm5_36h.

To evaluate Cm resistance stability in CTT liquid medium (without Cm addition), we performed a loss frequency test on CTT agar plates with no Cm. After the transfer of Cm5_36h cells to fresh medium lacking the antibiotic, cells were observed to grow as normal. However, it was found that the efficiency of growth on Cm agar dropped sharply after 5 h (approximately one generation) (Additional file 1: Fig. S1). The drop reflected the numbers of Cm-resistant cells. When phenotype Cm5_36h was grown in CTT + Cm liquid medium spread on CTT + Cm agar, the number of colony-forming units did not change, compared to wild type DK1622 in CTT medium. Besides the Cm5_36h and DK1622 wild phenotype at the mid-exponential stage in CTT liquid medium (the sample was abbreviated NDK), DK1622 induced by CTT + Cm at 8 h (in this early dormancy stage) was used to perform RNA sequencing, to avoid third-day dormancy, which may represent the spore-forming stage. The sample was abbreviated as Cm_8h.

### Identification of expressed transcripts and expression level analysis in the *M. xanthus* transcriptome

This characteristic is similar to that of *M. xanthus* FB_t_Cam_1_^r^, one of the spontaneous chloramphenicol-resistant isolates [19], which was reported due to the activity of Cm acetyltransferase from cell-free extracts. To understand drug-resistance mechanisms and to develop a new antibiotic screening method via homologous integration into chromosomes, genes correlated to the drug resistance were identified and a sensitive strain for chassis development was constructed. For each samples, 5,400,651 to 10,042,311 raw reads were generated. After removing adapter and ploy-N containing reads as well as low quality reads from raw data, 3,924,175 to 9,584,772 clean reads and total 7350 transcripts were obtained from the three samples (Additional file 2: Fig. S2 and Additional file 3: Table S1). The clean transcripts were used for further analysis. The percentage of genes having different expression levels were listed in Additional file 4: Table S2. Among 7348 predicted protein coding genes in the reference genome, 7094 (95.3% of the total genes, RPKM ≥ 15), 7257 (98.7% of the total genes, RPKM ≥ 15) and 7094 (96.5% of the total genes, RPKM ≥ 15) genes were detected in control, Cm_8h and Cm5_36h conditions, respectively (Additional file 4: Table S2). As can be seen from Additional file 5: Fig. S3 and Additional file 6: Fig. S4, the distribution of genes having different expression levels in Cm5_36h was similar to that of NDK before Cm-treatment. However, after treatment of the dormant strain cm8h, the number of genes at high expression levels was higher than in Cm5_36h, which indicated a strong response of genes to Cm treatment. Markedly, the percentage of FPKM (fragments per kilobase of transcript per million fragments mapped) > 60 is more than 55%, covering 4092 transcribed genes (Additional file 4: Table S2). The percentages of reads mapped to the reference genome in different samples were shown in Additional file 7: Table S3.

**Fig. 2:**
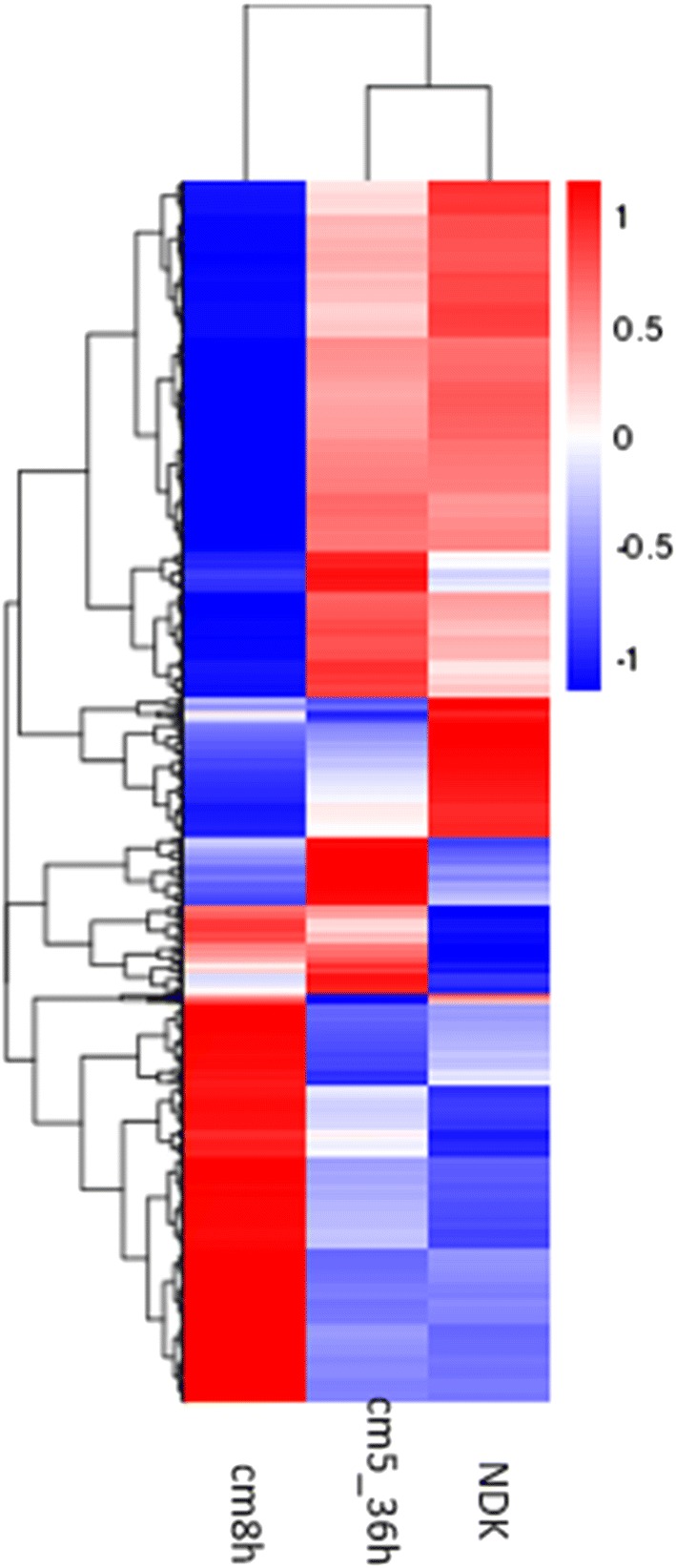
Cluster analysis of differentially expressed genes

Chromosomal distributions of the reads in three samples were shown in Additional file 8: Fig. S5. The density of Cm5_36h reads in the chromosome region was similar to that of NDK, showing that regions encoding secondary biosynthetic gene clusters were actively transcribed (Fig. 2). Conversely in Cm_8h, such regions were not actively transcribed (Additional file 8: Fig. S5).

Furthermore, the distribution of reads mapped to genomic regions were also investigated according gene structure, sequence coding for amino acids in protein (CDS) and intergenic regions. More than 80% of bases were mapped to CDS regions in NDK and Cm5_36h samples, and < 70% of bases were mapped to CDS regions in Cm_8h samples, which indicated that sample Cm_8h was experiencing an adapted period of the dormant phase (Additional file 5: Fig. S3).

### Identification of differentially expressed genes between different samples

To understand the mechanism of Cm resistance, differentially expressed genes (DEG) between different samples were analyzed by transcriptome studies. Comparison of the transcription levels of unigenes between Cm resistant Cm5_36h and Cm susceptible NDK revealed 481 DEGs, including 297 upregulated genes and 184 downregulated genes (Additional file 9: Fig. S6A). Comparison of Cm_8h and NDK identified 1920 DEGs, which included 625 upregulated and 1295 downregulated (Additional file 9: Fig. S6C) genes. Comparison of Cm5_36h to Cm_8h displayed 1970 DEGs, of which 1300 were upregulated and 670 downregulated (Additional file 9: Fig. S6B). Shown in a Venn diagram, a total of 167 DEGs were downregulated and 101 DEGs were upregulated in Cm5_36h and Cm_8h, in which 5 DEGs were upregulated in Cm5_36h, Cm_8h and Cm5_36h vs Cm_8h (Additional file 10: Fig. S7B). The total number of DEGs was also shown in a Venn diagram (Additional file 10: Fig. S7A).

### Functional distribution of differentially expressed genes

**Fig. 3:**
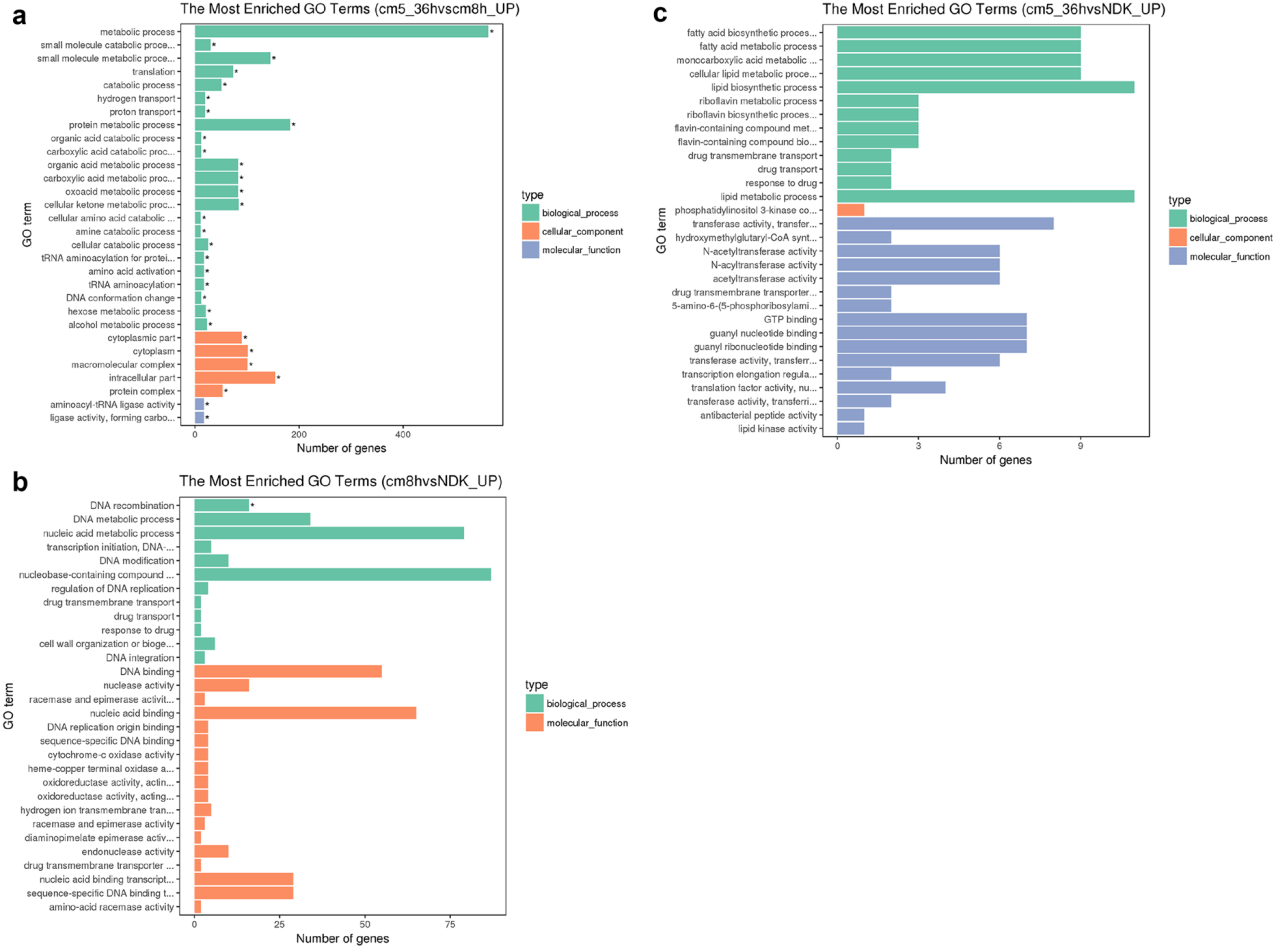
Comparison of functional annotations between different samples. **a** Comparable group: Cm5-36 and NDK; **b** comparable group: Cm_8h and NDK; **c** comparable group: Cm5_36h and Cm_8h. The green bars represent biological processes; orange bars represent cellular components; purple bars represent molecular functions

**Fig. 4:**
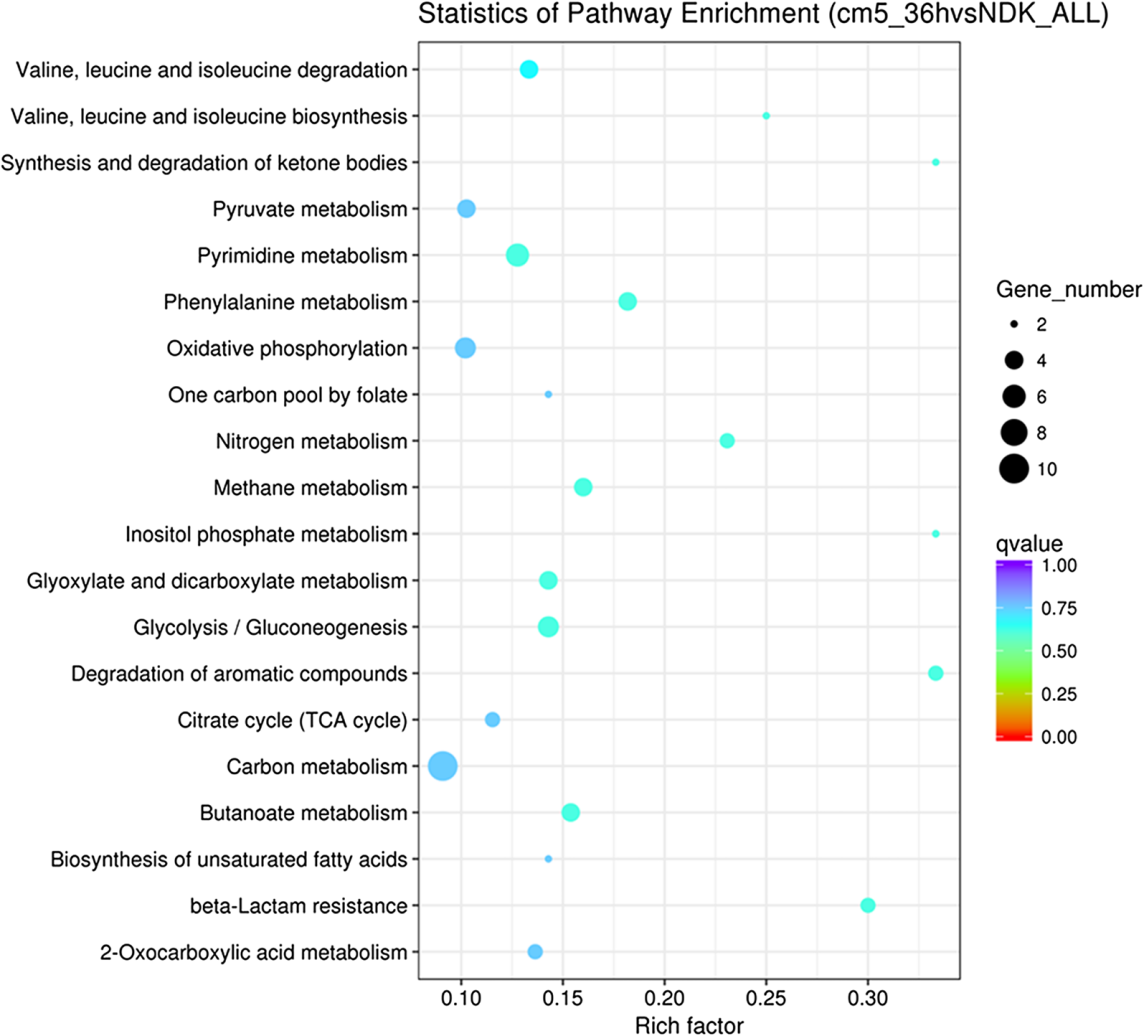
KEGG enrichment analysis of DEGs between Cm5_36h and NDK Y-axis label represents the distinct KEGG pathways, and X-axis label represents rich factor (rich factor = amount of DEGs in the pathway/amount of all genes in background gene set). The colors of the dots represent the Q-values of enrichment. Red color indicates high enrichment, while blue color indicates low enrichment. Pathway terms were sorted by Q-value in ascending order; and were marked in bold and underlined when Q-value < 0.05. The sizes of the dots represent the gene number of enrichments

According to GO (Gene ontology) categories, a comparison has been made representing the 30 major functional groups significantly upregulated in Cm5_36h vs Cm_8h (Fig. 3a). In Cm_8h vs NDK, there was only one group, DNA recombination, which was significantly upregulated, indicating that the cell was activated to accept foreign genes, such as those that confer antibiotic resistance (Fig. 3b). In Cm5_36h vs NDK, DE genes were also categorized into 30 major functional groups. However, no group was significantly upregulated (Fig. 3c). On the contrary, all of the 30 major functional groups were significantly upregulated in Cm5_36h vs Cm_8h. Although there were no significant differences in GO function for up-regulation in Cm5_36h vs NDK, we attempted to analyze the 30 top functional groups to elucidate an antibiotic resistance mechanism and related functional groups induced by the addition of Cm. These 30 top functional groups in Cm5_36h vs NDK mainly included transferase-related activity, drug-related transport and fatty-acid-related processes. The upregulated and downregulated groups in these top 30 major functional groups are listed in Additional file 11: Fig. S8. KEGG pathway enrichment in phenotype Cm5_36h was also analyzed, and beta-lactam resistance as well as the degradation of aromatic compounds which are possibly related to Cm resistance and degradation were observed (Fig. 4), suggesting multiple-drug resistance mechanisms might play an important role of Cm resistance.

### Some acetyltransferases were differentially expressed upon Cm induction

Some *M. xanthus* species can be induced to become Cm-resistant due to the existence of Cm acetyltransferase [19]. Among the 625 upregulated DEGs in Cm_8h vs NDK, seven genes (MXAN_0841, MXAN_1079, MXAN_1237, MXAN_2227, MXAN_3231, MXAN_4520, and MXAN_5479) encoding enzymes were found which might be involved in conferring Cm specific acetyltransferase activity. Similarly, among the 135 upregulated DEGs in the Cm5_36h vs NDK, six similar genes (MXAN_0841, TMXAN_1079, MXAN_1237, MXAN_4417, MXAN_6182 and MXAN_6984) were also found (Table 1).

**Table 1:** differentially expressed genes related with acetyltransferase in Cm_8h and Cm5_36h

Gene name	Description	log_2_FoldChange	Phenotype name
MXAN_0841	Glycine acetyltransferase	1.96	Cm5_36h
		2.48	Cm_8h
MXAN_1079	*N*-Acetyltransferase	1.58	Cm5_36h
		2.58	Cm_8h
MXAN_1237	*N*-Acetyltransferase	1.79	Cm5_36h
		1.47	Cm_8h
MXAN_4417	*N*-Acetyltransferase	1.35	Cm5_36h
MXAN_6182	*N*-Acetyltransferase	1.18	Cm5_36h
MXAN_6984	*N*-Acetyltransferase	1.82	Cm5_36h
MXAN_2227	*N*-Acetyltransferase	1.04	Cm_8h
MXAN_3231	*N*-Acetyltransferase	1.02	Cm_8h
MXAN_4520	*N*-Acetyltransferase	1.20	Cm_8h
MXAN_5479	Sugar *O*-acetyltransferase	1.38	Cm_8h

According to predictions for ARDB antibiotics based on the Resistance Genes Database, MXAN_5479 was predicted to belong to group B of Cm acetyltransferases, which can inactivate chloramphenicol through the addition of an acetyl group. Before we performed this transcriptome sequencing, we predicted that MXAN_5479 might be involved in Cm resistance, and deleted the gene. However, the mutant strain did not present any differences compared with the wild type DK1622 (data not shown). Although the transcription level of MXAN_5479 was upregulated in cm8h vs NDK (log2FC, 1.38), it was not changed in Cm5_36h vs NDK.

### Membrane-related proteins were upregulated in Cm induced culture

In the Cm5_36h vs NDK, MXAN_90 and MXAN_91 were observed to be related to drug transmembrane transport, drug transport and responses to drugs (at the level of biological processes) in KEGG pathway analysis. They were also related to drug transmembrane transporter activity (at the level of molecular functions). These two genes were also observed to be upregulated in Cm_8h vs NDK. They belong to a group of RND transporters, which is one of five main kinds of transporter related to antibiotics resistance. This operon was identified to be highly upregulated upon Cm induction in cm8h vs NDK and Cm5_36h vs NDK. To investigate the possibility of other transporters, we listed the MFS transporter, ABC transporter, and MATE transporter which were up-regulated in Cm_8h vs NDK and in Cm5_36h vs NDK.

Markedly, among the DEGs in the Cm-resistant strain Cm5_36h compared to NDK, the MFS transporter (MXAN_2566) was found to be the most highly upregulated gene (log2FC, 5.60), which was annotated to be a Cm resistance gene (CmlA/FloR family chloramphenicol efflux MFS transporter). The MATE family transporter (MXAN_7022) was the second upregulated transporter (log2FC, 3.00) in Cm5_36h. However, among the DEGs in Cm_8h, the RND transporter (MXAN_0091) mentioned above was the most upregulated gene (log2FC, 4.65), and the MATE family transporter (MXAN_7022) was the second upregulated (log2FC, 3.92) (Table 2).

**Table 2:** differentially expressed genes related with transporter in Cm_8h and Cm5_36h

Gene name	Description	log_2_FoldChange	Phenotype name
MXAN_2566	MFS transporter	5.60	Cm5_36h
		1.42	Cm_8h
MXAN_7022	MATE family transporter	3.00	Cm5_36h
		3.92	Cm_8h
MXAN_0091	RND transporter	2.32	Cm5_36h
		4.65	Cm_8h
MXAN_0092	RND transporter	1.46	Cm5_36h
		1.38	Cm_8h
MXAN_7119	MATE family transporter	1.12	Cm5_36h
		1.01	Cm_8h
MXAN_1286	ABC transporter	1.57	Cm5_36h
MXAN_6403	ABC transporter	1.08	Cm5_36h
MXAN_0819	Biopolymer transporter	1.61	Cm5_36h
		2.19	Cm_8h
MXAN_2948	ABC transporter	1.14	Cm_8h
MXAN_5963	MFS transporter	1.27	Cm_8h
MXAN_0274	Biopolymer transporter	1.14	Cm_8h
MXAN_3225	ABC transporter	1.04	Cm_8h
MXAN_3650	ABC transporter	1.13	Cm_8h
MXAN_3677	MFS transporter	1.12	Cm_8h
MXAN_4623	ABC transporter	3.18	Cm_8h
MXAN_4818	ABC transporter	1.01	Cm_8h
MXAN_4819	ABC transporter	1.07	Cm_8h
MXAN_5186	Multidrug transporter	1.13	Cm_8h

MXAN_0091, MXAN_0092, MXAN_0819, MXAN_2566, MXAN_7022, MXAN_7119 were simultaneously upregulated in Cm_8h vs NDK and Cm5_36h vs NDK indicating that they are all related to the Cm resistance.

To further verify the RNA sequencing data, we investigated the transcription pattern of MXAN_2566 of DK1622 in CTT + Cm liquid medium by real time PCR, and found that there was a sharply increased transcription level at the first and last stages, but in the middle stage the transcription level of MXAN_2566 was reduced (Additional file 12: Fig. S9).

### DEGs involved into secondary biosynthetic gene clusters

MXAN_3462, MXAN_3932, MXAN_3933, MXAN_3935, MXAN_3936, MXAN_3938, MXAN_3941, MXAN_4301 and MXAN_6401 located in the gene clusters of primary and secondary fatty acid biosynthesis were significantly upregulated in Cm5-36h (Additional file 13: Table S4). 6 out of 9 genes (MXAN_3932, MXAN_3933, MXAN_3935, MXAN_3936, MXAN_3938, MXAN_3941) were located in secondary metabolite TA clusters. The other three genes were located in other secondary biosynthetic gene clusters: MXAN_3462 was found in an unidentified myxalamid, MXAN_4301 in a yellow pigment DKxanthene cluster (MXAN_4290 to MXAN_4305), and MXAN_6401 in a lantibiotics cluster. Interestingly, all 20 TA genes (from MXAN3931 to MXAN3950) were upregulated, which suggested that TA biosynthesis was positively related to Cm resistance. Besides the TA gene cluster, the Lichenicidin/Mersacidin biosynthetic gene cluster, belonging to lantipeptide-t2pks, contained 34 genes from MXAN_6380 to MXAN_6413, where 7 genes were upregulated and none of genes were downregulated (Additional file 14: Table S5). On the contrary, some genes in the other clusters were downregulated, for example, in well studied myxalamid gene cluster (MXAN_4525 to MXAN_4530), 2 out of 5 genes were downregulated. The gene expression level of Myxochromide (MXAN_4077 to MXAN_4079), myxochelin (MXAN_3647 to MXAN_3640) and myxoprincomide (MXAN_3779) gene clusters were not changed in Cm5_36h vs NDK.

The transcription levels of TA clusters Dkxanthene, Myxalamid, Myxochromide, myxochelin, and myxoprincomide were not changed in Cm_8h vs NDK. MXAN_3462, located in an unknown myxalamid cluster, was up-regulated (log2FC, 1.69). Of the related DEGs in the type 2 lantibiotic biosynthetic gene cluster in cm8h, only MXAN_6392 (an acyl carrier protein) and MXAN_6393 (a 3-hydroxyacyl-[acyl-carrier-protein] dehydratase FabZ) were up-regulated (log2FC, 2.07, and 1.32, respectively).

### DEGs involved in unfolded protein degradation caused by Cm addition

Cm can inhibit protein biosynthesis by binding to ribosome to influence the production of proteins. We found that MXAN_4823, MXAN_4824 and MXAN_4825, which consist of one operon encoding the ATP-dependent Clp protease ATP-binding subunit were significantly upregulated (log2FC 1.47, 2.17, 1.92) in Cm5_36h vs NDK. The three genes also were upregulated in cm8h vs NDK (log2FC 3.57, 2.67, 2.61). MXAN_4832 ATP-dependent Clp protease ATP-binding subunit (log2FC 1.19) was also upregulated in Cm_8h vs NDK. ClpA is an ATP-dependent chaperone and part of the ClpAP protease that participates in regulatory protein degradation and the dissolution and degradation of protein aggregates. In both Cm_8h and Cm5_36h vs NDK, these proteases were all upregulated, which might be related to the degradation of unfolded proteins because of Cm-induced functionality disorder of 30S rRNA.

Since the growth rate of the Cm5_36h cells was fast, the transcription of some tRNAs and rRNA were significantly increased, for example, Log2FC of tRNA-Tyr, tRNA-Gly, tRNA-Arg, tRNA-Leu and 23S ribosomal RNA was 5.28, 5.27, 4.60, 2.94 and 3.98 respectively (Additional file 15: Table S6).

### DEGs related to growth arrest, cell cycle, and stress in dormant phenotype Cm_8h

To understand why Cm_8h has a long lag growth phase and to elucidate the molecular activity of bacteria at this stage, we investigated upregulated genes in Cm_8h vs NDK, where the number of downregulated genes (1295 genes) was larger than that of the upregulated genes (625 genes). DK1622 is a well-studied bacterial strain with a complex lifecycle and forms fruiting-bodies on agar plates under starvation conditions. During growth arrest in CTT + Cm, overall gene expression activity was much lower than that of the exponential phase or the transition to stationary phase. In the transition to stationary phase, RelA can be activated to synthesize alarmone guanosine pentaphosphate (ppGpp), which represses the transcription of rRNA and leads to a redirection of biosynthetic capacity toward more urgent concerns, such as the prevention or repair of DNA damage, or alternatively to osmoprotection. Simultaneously, housekeeping sigma factor RpoD (MXAN_5204) was inhibited (-2.69) and some other RNA polymerase sigma factors were activated in response to this induction, for example, MXAN_0233 (log2FC,1.28), MXAN_2184 (log2FC,1.76), MXAN_4987 (log2FC,2.38). However, we did not observe a change in RelA gene (MXAN_3204) transcription. Genes related to chromosome partition, such as MXAN_7477, were also down regulated (log2FC, -2.15). Genes related to chromosome maintenance and DNA damage were highly activated, for example, DNA starvation/stationary phase protection protein Dps (MXAN_1562 log2FC 2.33), HNH endonuclease (MXAN_1486 log2FC 8.09, MXAN_6077 log2FC 2.14), and virulence-associated protein E domain protein (MXAN_1209 1.98).

### Knockout of gene MXAN_2566 and its comparison with wild type

**Fig. 5:**
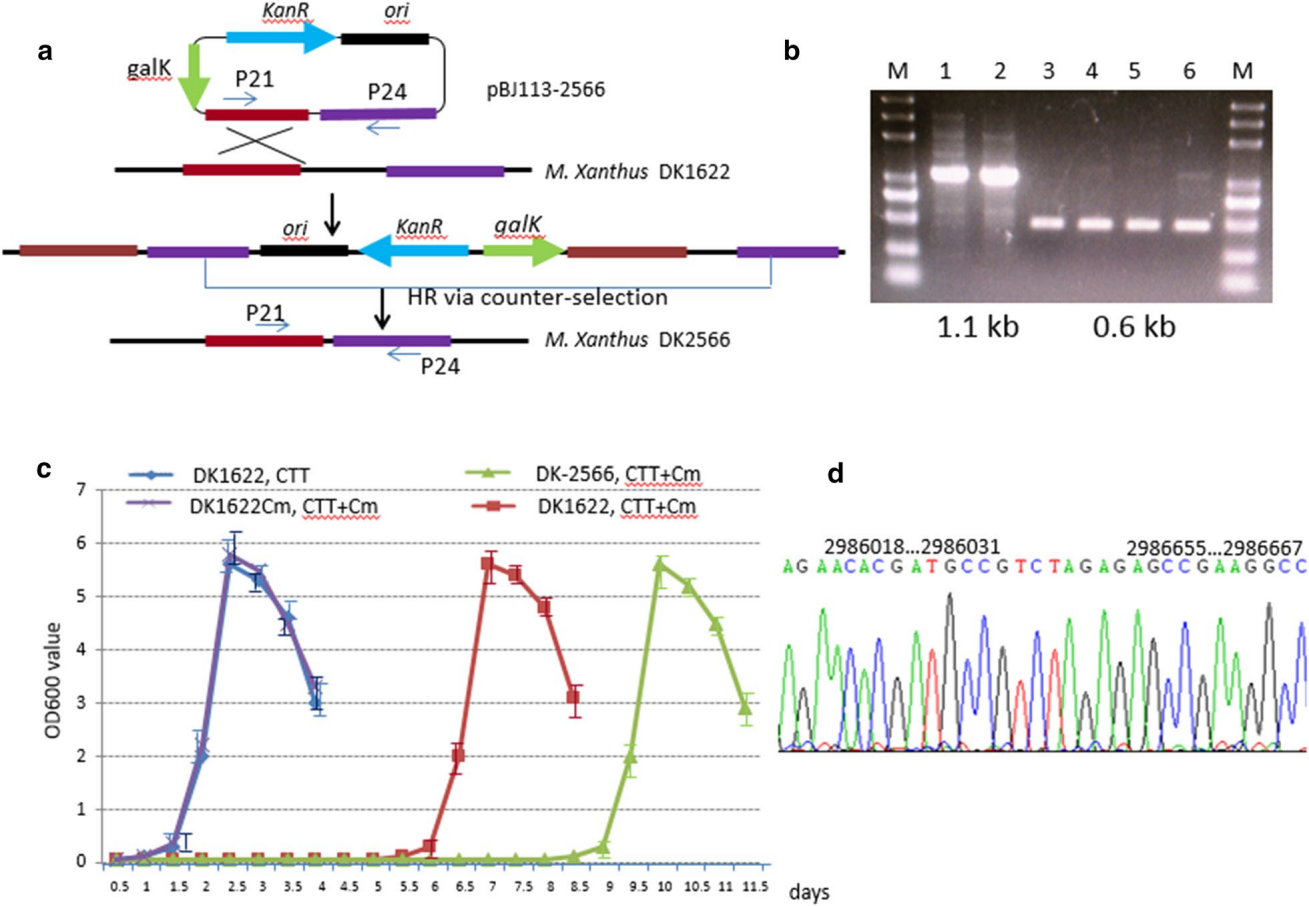
Deletion of drug resistant gene MXAN_2566 in *M.xanthus* DK1622. **a** Schematic diagram to delete MXAN_2566 in DK1622 Firstly, pBJ113-2566, which contains two homologous arms, was integrated into the genome of DK1622 by kanamycin selection, and the second crossover occurred by galactose-addition to the culture to obtain strain DK-2566. **b** Identification of double crossover strains DK-2566 by agarose gel electrophoresis of PCR product. Lanes 1 and 2 are the bands from wild type DK1622 (1.1 kb), lanes 3, 4, 5, and 6 show 0.6 kb bands representing the double crossover strain DK-2566 using primer p21 and p24. **c** Growth curves of *M. xanthus* wild type and mutant strains in CTT liquid medium with or without Cm. DK1622Cm indicated the DK1622 already adapted in Cm-containing CTT + Cm liquid medium, accordingly the growth curve of DK1622Cm/CTT + Cm is similar to that of DK1622 in CTT liquid medium. Fresh DK1622 wild type would stay 6 days dormancy in CTT + Cm liquid medium, which has a dormant phase. While drug resistance gene deleted strain DK-2566 stays 9 days dormancy, which is beyond the colony growth time (7 days) on CTT agar plate. 5D. Chromatograms of the 0.6-kb PCR product DNA sequence. The PCR primers P21, P24 as the sequencing primers were used to confirm the deleted fragment. The nucleotide position of the flanking regions between the Restriction enzyme site *Xba*I (TCTAGA) were marked on the peak of chromatograms

To verify the prediction that MXAN_2566 was the main player in Cm resistance in DK1622, we knocked out the gene via homologous recombination using kanamycin selection and galactose counter-selection (Fig. 5a). Full length MXAN_2566 is 1235-bp, and it was annotated to be a drug resistance MFS transporter belonging to the Bcr/CflA family protein. We obtained strain DK-2566 where the 570-bp interior region was deleted by a PCR method and sequenced (Fig. 5d). Compared to the wild type strain, a 0.6-kb band appeared in the mutant type strain DK-2566. There was 1.1-kb band in wild type DK1622 (Fig. 5b). Strain DK-2566 was not grown for 7 days in CTT + Cm liquid medium, but its short growth time was enough to isolate the subsequent mutant strain by Cm screening (Fig. 5c). We did not observe any difference in the growth curve or doubling time in CTT liquid medium between DK-2566 and wild type DK1622 without Cm supplement. On CTT agar plates, DK-2566 has the same ability to form yellow or tan color colonies as wild type DK1622.

### Integration of homologous arms into the genome via Cm selection

**Fig. 6:**
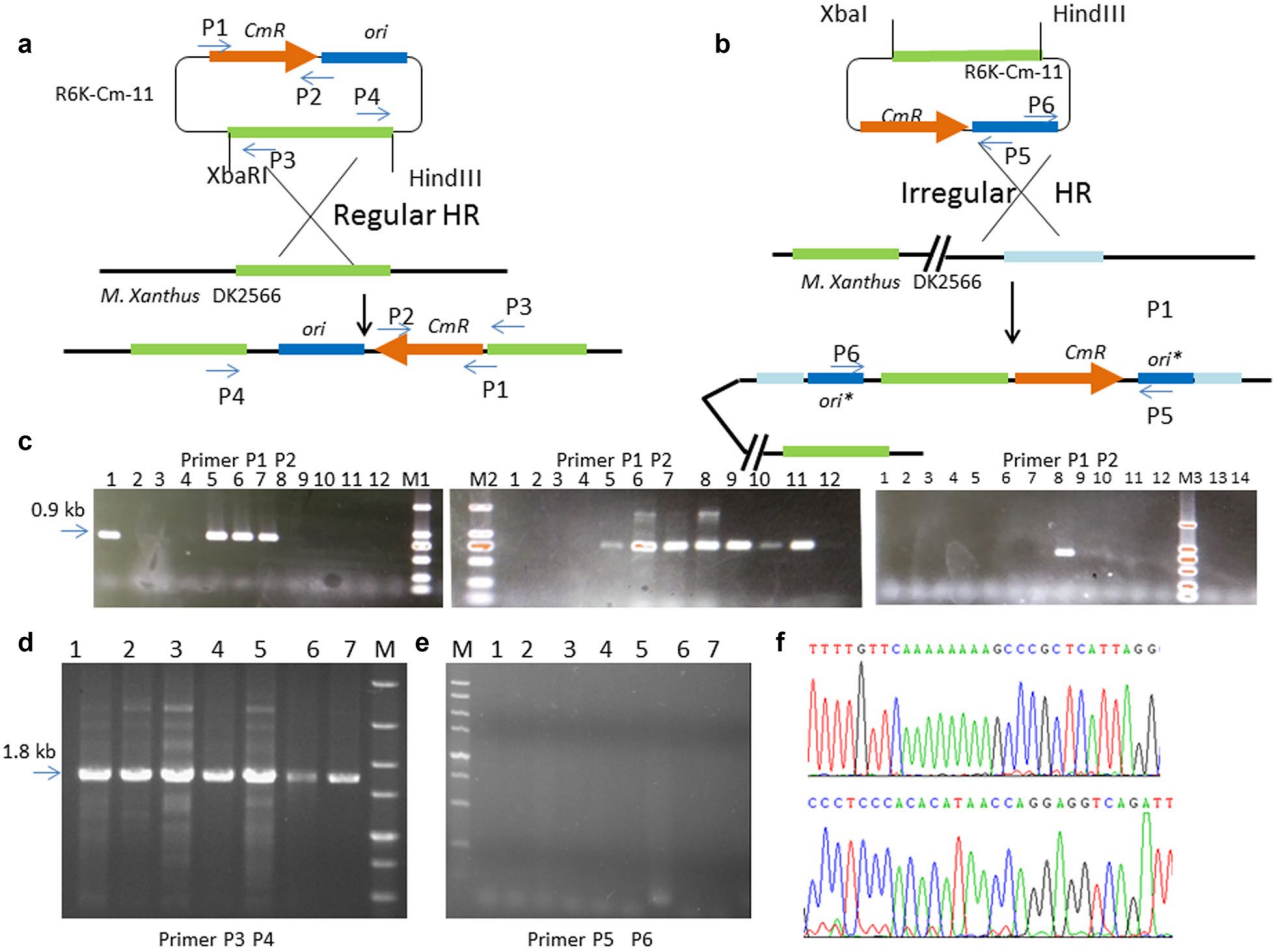
Integration of homologous recombination by Cm selection in *M. xanthus* DK-2566. **a** Schematic diagram of the integration into the genome of DK-2566 via regular homologous recombination by pR6K-Cm-11. Cm-positive clones were further investigated by PCR with primers (marked in P1 and P2) designed onto the Cm resistant gene CmR (shown in orange). **b** Schematic diagram of the irregular integration into the genome of DK-2566 via irregular homologous recombination by pR6K-Cm-11. Firstly, suicide plasmid pR6K-cm-11 was electro-transformed into the cell of DK-2566. **c** Identification of integration into the genome of DK-2566 by agarose gel electrophoresis of PCR products in Cm-positive clones. Lane M1-1,-5,-6,-7, M2-5, -6, -7, -8, -9, -10, -11, M3-8 show 0.9 kb bands showing CmR gene of strains DK-2566-11 by primer P1 and P2. No band in other lanes was amplified, although these clones can grow in a Cm agar plate. **d** Identification of correct integration into the genome of DK-2566 was performed using agarose gel electrophoresis of PCR products of Cm-positive clones. Primers (marked in P3 and P4) were designed to target both ends of homologous arm (shown in light green). Lanes 1–7 show 1.8 kb bands of the vector region integrated into the genome in strain DK-2566-11 by primer pairs P3 and P4. **e** Verification of irregular integration occurring at the region of the vector was assessed by agarose gel electrophoresis. Primers pairs P5 and P6 located in the backbone were designed to verify that the recombination did not occur at the vector region. **f** Chromatograms of the 1.8 kb PCR product amplified and sequenced using primers P3 and P4 as sequencing primers

To verify whether strain DK-2566 can be used as a chassis to perform genetic operations by Cm selection, we constructed a series of plasmids contained homologous arms from DK1622 and attempted to integrate the homologous arm into DK-2566. Plasmids pR6K-Cm-11 and pR6K-Cm-14 was electro-transformed into DK-2566. After several round of purification on CTT + Cm agar plates (Fig. 6a), we performed colony PCR to identify mutant strains containing integrated plasmids by primer pairs 719f and 1534r, which are located in the *cat* gene regions. As shown in Fig. 6c, 0.8-kb bands were present. Although we obtained mutant strains resistant to Cm (10 μg/ml), not all of the strains contained the 0.8-kb *cat* gene fragment amplified by the primer pairs (Fig. 6c). To investigate whether the concentration of Cm affected integration efficiency by Cm selection, we performed a Cm selection experiment on CTT + Cm agar plates containing 8, 12, 16 μg/ml of Cm. We found that about 21% integration efficiency was achieved regardless of the concentration of Cm, and higher concentrations did not increase it (Table 3).

**Table 3:** Comparison of integration efficiency via homologous recombination by pR6K-Cm-11after screening at different Cm concentration in DK-2566

Strain	Cm (μg/ml)	Number of Cm resistance (three replication)	Number of PCR positive by primer P1 and P2 per 100 colony			PCR positive by primer P3 and P4 (%)	PCR positive by primer P5 and P6 (%)
DK1622	10	100	0	0	0	100	0
DK-2566	8	100	19	21	22	100	0
	12	100	19	20	23	100	0
	16	100	18	16	25	100	0

To confirm whether the integration occurred at the correct position for the homologous arm in the chromosome, we designed two primer pairs, P3 and P4 to amplify the entire vector sequence (Fig. 6a). PCR bands at size 1.8 kb were present in all of the *Cat* fragment positive colonies (Fig. 6d), and PCR products were then verified by sequencing (Fig. 6f). These results directly indicate that homologous recombination occurred at the correct position, at around 1.0 kb on the homologous arm. To further prove the correct integrational position, we also designed another PCR primer pair, P5 and P6, located in the region of R6K *ori* as counter-evidence and did not obtain any bands, indirectly verifying the integration position (Fig. 6e).

## Discussion

### Acetyltransferases might be involved in Cm resistance in *M. xanthus*

Transfer of drug resistance to *Myxococcus* from other bacteria carrying drug-resistance R factors, or from *Escherichia coli* phage P1 have already been reported [22, 23]. Cell-free preparations of Cm-resistant strains can catalyze the acetylation of Cm [19]. A bacteriophage from *Escherichia coli* can inject its DNA into *M. xanthus* despite the wide taxonomic gap between *Myxococcus* and *E. coli* carrying a gene for Cm resistance into *M. xanthus*, generating unstable antibiotic-resistant strains [23].

Before we performed RNA sequencing, we presumed that acetyltransferases might be the main factor affecting resistance to Cm and that MXAN_5479 might therefore dominate in Cm resistant strains based on the literature, and predictions made on the whole genome by resistance prediction software. However, we did not observe differences in growth curves upon deletion of MXAN_5479. According to RNA sequencing results, the MXAN_5479 gene was upregulated in cm8h vs NDK, but we did not find any change in transcription levels of MXAN_5479 in cm5_36h vs NDK.

### Membrane transporters are involved in drug resistance

MXAN_2566 was the most highly upregulated gene found in Cm resistant phenotype cm5_36h, which belongs to the MFS family of transporters. MXAN_2566 was annotated to be a Cm resistance gene (CmlA/FloR family Cm efflux MFS transporter). After deletion of MXAN_2566, we successfully constructed mutant strain DK-2566 which cannot be grown in CTT + Cm medium. In contrast, MXAN_3444 was annotated to be a tetracycline resistant MFS efflux pump. However, even if MXAN_3444 exists, the *tetR* gene [*tet* (C)], which belongs to the MFS_1 superfamily, is often used in genetic operation of *M. xanthus* DK1622, for example in site-specific integration plasmid pSWU30 and homologous recombination plasmid pNG10 [9].

Multidrug transporters play an important role in the excretion of toxic compounds, occasionally also including specific antimicrobial agents from bacterial cells. Multidrug transporters in bacteria fall into five distinct protein super families, namely the ATP binding cassette super family (ABC), major facilitator super family (MFS), small multidrug resistance super family (SMR), multidrug and toxic compound extrusion (MATE) super family, or the resistance-nodulation-cell division (RND) super family. In the Cm5_36h phenotype, MATE family transporters (MXAN_7022), RND transporters (MXAN_0091), ABC transporters (MXAN_1286), RND transporters (MXAN_0092) are the second, third, fourth and fifth highest upregulated, respectively. However, in the Cm-resistant strain Cm_8h vs NDK, an RND transporter (MXAN_0091) was found to be the most highly upregulated gene (log2FC, 4.65) while a MATE family transporter (MXAN_7022), an ABC transporter (MXAN_4623), and an MFS transporter (MXAN_2566) were the second, third and fourth highly upregulated in Cm_8h vs NDK. We do not know why MXAN_2566 is not the most highly upregulated in the lag phase growth of Cm_8h vs NDK, which was observed as the mostly highly upregulated in Cm5_36h. Therefore, considering the growth curve, we preferentially choose MXAN_2566 (log2FC, 5.60) as a candidate gene for the main resistance factor against Cm in Cm5_36h.

MXAN_0091, MXAN_0092, MXAN_0819, MXAN_2566, MXAN_7022, and MXAN_7119 were simultaneously upregulated in Cm_8h and Cm5_36h cells, indicating that they were all related to the resistance of Cm. This may be the reason why strain DK-2566 is able to remain dormant for 9 days and grow up after the dormancy stage. Numerous upregulated transporters and acetyltransferases explain why we obtained only 21% efficiency for positive colonies in CTT + Cm plate, which indicated there would be a complex mechanism of Cm resistence in *M. xanthus*.

## Conclusions

In this study, we report the induction and characterization of a Cm-resistant phenotype and a Cm-dormant phenotype, compared to a Cm-sensitive phenotype (as a control), based on Illumina RNA sequencing in *M. xanthus* DK1622. In disagreement with reports published 40 years ago, we found that a major facilitating superfamily transporter, not Cm acetyltransferase, played a key role in conferring resistance to Cm in *M. xanthus*. To broaden the spectrum of available molecular tools, we developed an alternative selection system based on Cm by genetic knockout and chromosome integration via homologous recombination. This study provides a new antibiotic selection system and makes steps towards understanding this antibiotic resistance mechanism in *M. xanthus* DK1622.

## Materials and methods

### Bacterial strains, growth conditions, plasmids, and oligonucleotides

*Myxococcus xanthus* and *E. coli* strains used in this study are listed in Table 4. *M. xanthus* strains were grown at 30 °C in liquid Casitone-Tris (CTT) medium with vigorous shaking. When necessary, media were supplemented with kanamycin (40 µg/ml), tetracycline (15 µg/ml), or chloramphenicol (10 µg/ml). *E. coli* strains were grown at 37 °C in Luria–Bertani medium, which was supplemented with ampicillin (100 µg/ml), kanamycin (20 µg/ml), tetracycline (10 µg/ml) or chloramphenicol (10 µg/ml) when needed. The plasmids used in this study are listed in Table 5. DNA manipulation and cloning were performed according to the standard protocols in Molecular Cloning: A Laboratory Manual (Third Edition) [24]. Laboratory constructed plasmids were verified by PCR and DNA sequencing. Oligonucleotides used in this study were listed in Additional file 16: Table S7.

**Table 4:** Strains used in this study

	Genotype	Reference
Strains		
*E. coli* TOP10	F–*mcr*A Δ(*mrr*-*hsd*RMS-*mcr*BC) Φ80*lac*ZΔM15 Δ*lac*X74 *rec*A1 *ara*D139 Δ(*araleu*) 7697 *gal*U*gal*K*rps*L (StrR) *end*A1 *nup*G	Life technologies
*E. coli* GBdir-pir116	DH10B, fhuA::IS2, ΔybcC, ΔrecET, pir116) with an arabinose-inducible ETγA operon (full-length recE, recT, redγ and recA), and a copy-up pir116 gene in chromosome); this allows the replication of R6K plasmids in high copy number	Wang et al. [33]
*M. xanthus*		
DK1622	Wild type	Kaiser [34]
DK-5479	DK1622 ΔMXAN_5479	This study
DK-2566	DK1622 ΔMXAN_2566	This study
DK-Cm-11	DK-2566∷pR6K-Cm-11	This study
DK-Cm-14	DK-2566∷pR6K-Cm-14	This study

**Table 5:** Plasmids used in this study

Name	Description	Reference or source
pBJ113	pBR322 *ori*,*Gal*K; Kan^R^	Julien et al. [32]
pR6K-Cm-ccdB	R6K *ori*, Cm^R^,	Wang et al. [33]
pBJ113-5479Up	Kan^R^, one homologous arm of MXAN_5479 flanked by *Eco*RI + *Xba*I	This study
pBJ113-5479	KanR, two homologous arms of MXAN_5479 flanked by *Eco*RI + *Hin*dIII	This study
pBJ113-2566Up	KanR, one homologous arm of MXAN_2566 flanked by *Eco*RI + *Xba*I	This study
pBJ113-2566	KanR, two homologous arms of MXAN_2566 flanked by *Eco*RI + *Hin*dIII	This study
pR6K-Cm	CmR, R6K ori which can not replicate in normal *E.coli*	This study
pR6K-Cm-11	CmR, contains 1.9-kb homologous arm located in TA gene cluster	This study
pR6K-Cm-14	CmR, contains 1.6-kb homologous arm located in yellow pigment gene cluster	This study

### Sample collection and RNA preparation

Total RNA was extracted using TRIzol^®^ Reagent (Invitrogen) according to the manufacturer’s protocol. Three independent biological repeats were conducted for each treatment and extracted RNA samples were mixed together for each treatment. All RNA samples were treated with DNase I (TaKara, Dalian, China). RNA purity was checked using the NanoPhotometer^®^ spectrophotometer (IMPLEN, CA, USA). RNA concentration was measured using the Qubit^®^ RNA Assay Kit in a Qubit^®^ 2.0 Flurometer (Life Technologies, CA, USA). RNA integrity was assessed using the RNA Nano 6000 Assay Kit of the Bioanalyzer 2100 system (Agilent Technologies, CA, USA).

### Library preparation for Transcriptome sequencing

A total amount of 5 μg RNA per sample was used as input material for the RNA sample preparations. Sequencing libraries were generated using Illumina^®^ TruSeq^®^ Stranded Total RNA Sample Preparation kit. The first step involves the removal of ribosomal RNA (rRNA) with Ribo-Zero rRNA removal beads. Following purification, the RNA is fragmented into small pieces. The cleaved RNA fragments are copied into the first cDNA strand using reverse transcriptase and random primers. Strand specificity is achieved by replacing dTTP with dUTP in the second strand marking mix (SMM), followed by second strand cDNA synthesis using DNA Polymerase I and RNase H. Remaining overhangs were converted into blunt ends via exonuclease/polymerase activities. After adenylation of 3′ ends of DNA fragments, adaptors with hairpin loop structures were ligated to prepare for hybridization. To select cDNA fragments of preferentially 150–200 bp in length, the library fragments were purified with 2% Low Range Ultra Agarose. The products were enriched with PCR amplification using Phusion DNA polymerase (NEB) for 15 PCR cycles to create the final cDNA library. Library quality was assessed on the Agilent Bioanalyzer 2100 system.

The clustering of the index-coded samples was performed on a cBot Cluster Generation System using TruSeq PE Cluster Kit v3-cBot-HS (Illumia) according to the manufacturer’s instructions. After cluster generation, the library preparations were sequenced on an Illumina Hiseq 2000 Truseq SBS Kit v3-HS.

### Reads mapping to the reference genome

Raw data (raw reads) in fastq format were first processed by Trimmomatic with default parameters [25]. Clean data (clean reads) were obtained by removing reads containing adaptors, reads containing poly-N and low quality reads from raw data. At the same time, Q20, Q30 and GC content of the clean data were calculated. All downstream analyses were based on clean data of high quality based on these metrics.

Reference genome and gene model annotation files were directly downloaded from the genome website, with NCBI Reference Sequence: NC_008095.1. Index of the reference genome was built using Bowtie v2.0.6 while paired-end clean reads were aligned to the reference genome using TopHat v2.0.9 [26]. We selected TopHat as the mapping tool as it can generate a database of splice junctions based on gene model annotation files and thus a better mapping result than other non-splice mapping tools. Reads from all sequencing experiments are deposited under accession numbers SRX4504360 for sample NDK, SRX5472518 for sample Cm5-36h, and SRX5472519 for sample Cm_8h at the Sequence Read Archive in the NCBI homepage.

### Differential expression analysis and Functional enrichment

HTSeq v0.5.4p3 was used to count the read numbers mapped to each gene [27] and further, the reads per kilobase of exon model per million reads (RPKM) of each gene was calculated according to the length of the gene and read counts mapped to this gene. RPKM considers the effect of sequencing depth and at the same time the gene length for the read count, and is currently the most commonly used method for estimating gene expression levels. EdgeR was used for differential expression analysis [28]. The DEGs between two samples were selected using the following criteria for significantly differential expression: (i) absolute value of log2 (fold change) was greater than 1 and (ii) the false discovery rate (FDR) should be < 0.05. To understand the functions of the differentially expressed genes, GO functional enrichment and KEGG pathway analysis were carried out using Goatools and KOBAS respectively [29, 30]. DEGs were significantly enriched in GO terms and metabolic pathways when their Bonferroni-corrected P-value was < 0.05.

### Construction of Homologous arms plasmids pBJ113-2566

To construct the suicide plasmid pBJ113-2566, we firstly constructed pBJ113-Up2566 using a method which has been described previously [31]. First, we amplified the homologous arm using primer pairs DRT-356F and DRT-1280R, which contain *Eco*RI and *Xba*I, respectively. Then, we double digested it using *Eco*RI and *Xba*I, and ligated to linearized pBJ113 [32] with *EcoR*I + *Xba*I. After confirmation of pBJ113-Up2566 by enzymatic digestion and sequencing, it was linearized using *Xba*I and *Hin*dIII and ligated with the downstream homologous arm which was amplified using primer pairs DRT-1904F and DRT-1904F. This was then double digested using *Xba*I + *Hin*dIII, to give plasmid pBJ113-2566. Finally, the clones were confirmed by double molecular digestion and gene sequencing.

### Construction of vector pR6K-cm and homologous recombination plasmids pR6K-Cm-11, pR6K-Cm-14

To completely avoid the occurrence of the homologous recombination between the pBR322 derived origin region, we constructed a vector which contains a different ori R6K. Plasmid pR6K-Cm-ccdB and *E.coli* GBdir-pir116 [33] were used to construct plasmid pR6K-Cm by primer pairs XE307F, H-1534R and self-ligation. Plasmid pR6k-Cm was double digested by *Xba*I and *Hin*dIII and ligated with the digested fragment, amplified with primer pairs S11D-141f, S11D-2093R and S14D-1057f, S14D-2649R to construct plasmid pR6K-Cm-11, and pR6K-Cm-14.

### Construction of mutant strain DK-2566 and homologous recombination by Cm selection in DK-2566

To obtain the MFS deletion strain, we used the suicide plasmid pBJ113-2566 which contained two homologous arms to integrate into the genome of DK1622 [34] by kanamycin selection. After confirming integration by PCR amplification, the double crossover occurred on CTT + galactose agar plates. Correct recombinant colonies were confirmed by PCR amplification as well as by gene sequencing.

By the same method, we transformed the plasmid pR6K-cm-11 and pR6K-cm-14 into the DK-2566 strain. Strains DK-2566-cm11 and DK-2566-cm14 were obtained by Cm selection using different concentrations of CTT + Cm agar plates, and were confirmed by PCR amplification and sequencing.

## Additional files

**Additional file 1: Fig. S1.** Loss of Cm resistance by *M. xanthus* DK1622.

**Additional file 2: Fig. S2.** Classification of Raw Reads of three samples (A) Classification of Raw Reads of Cm5_36h; (B) Classification of Raw Reads of Cm_8h; (C) Classification of Raw Reads of NDK.

**Additional file 3: Table S1.** Transcriptome data output of three samples.

**Additional file 4: Table S2.** Percentages of genes in different expression levels.

**Additional file 5: Fig. S3.** Distribution of reads mapped to genomic regions of three samples. (A) Distribution of reads mapped to genomic regions in Cm5_36h; (B) Distribution of reads mapped to genomic regions in Cm_8h; (C) Distribution of reads mapped to genomic regions in NDK.

**Additional file 6: Fig. S4.** RPKM distribution for all samples.

**Additional file 7: Table S3.** Percentages of reads mapping to the reference genome.

**Additional file 8: Fig. S5.** Read density in the chromosomes of three samples (A) Read density of Cm5_36h in chromosomes; (B) Read density of Cm_8h in chromosomes; (C) Read density of NDK in chromosomes; (D) Genome map determined in Ref. [2]: layer 4 represents the biosynthetic gene cluster of secondary metabolites.

**Additional file 9: Fig. S6.** The overall distribution of DEGs in three samples.

**Additional file 10: Fig. S7.** Comparisons of the number and overlapping relationships of DEGs between different samples. A purple circle represents number of DEGs between Cm5_36h and Cm_8h; yellow circle stand for number of DEGs between Cm5_36h and NDK. The overlapping region means shared DEGs of two comparable groups. B. Purple circle represents number of DEGs between Cm5_36h and Cm_8h; yellow circle stand for number of DEGs between cm8h and NDK. The overlapping region means shared DEGs between two comparable groups. C. Purple circle represents number of DEGs between Cm_8h and NDK; yellow circle represents the number of DEGs between Cm5_36h and NDK. The overlapping region means shared DEGs between two comparable groups.

**Additional file 11: Fig. S8.** The most enriched GO terms between Cm5_36h and NDK.

**Additional file 12: Fig. S9.** The relative transcription level of MXAN_2566 of DK1622 in CTT + Cm by real time PCR using the same cell density.

**Additional file 13: Table S4.** Differentially expressed genes related with fatty acid process in Cm5_36h.

**Additional file 14: Table S5.** Differentially expressed genes related with type 2 lantibiotic biosynthetic gene clusters in Cm5-36h.

**Additional file 15: Table S6.** Differentially expressed genes related with protein biosynthesis in Cm5_36h.

**Additional file 16: Table S7.** Primers used in this study.

## Data Availability

All data generated or analyzed during this study are included in this published article and in its additional files.
