# Supplementary figures and images for: Whole transcriptome analysis and gene deletion to understand the chloramphenicol resistance mechanism and develop a screening method for homologous recombination in Myxococcus xanthus
Source: Microb Cell Fact. 2019 Jul 10;18:123. doi: 10.1186/s12934-019-1172-3 (PMC6617876; doi:10.1186/s12934-019-1172-3)

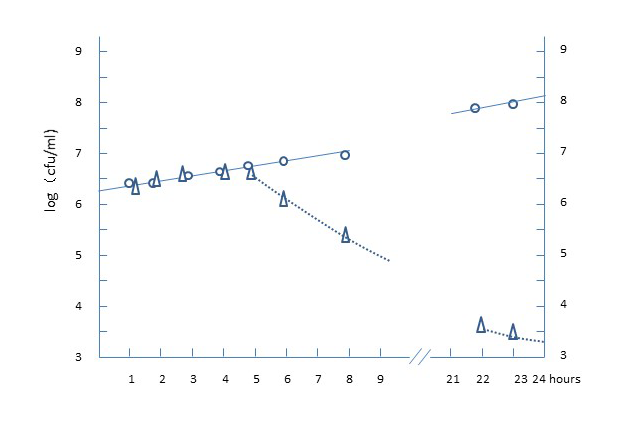

Supplement: Supplementary file 1 — Additional file 1: Fig. S1. Loss of Cm resistance by M. xanthus DK1622. [file 12934_2019_1172_MOESM1_ESM.tif]

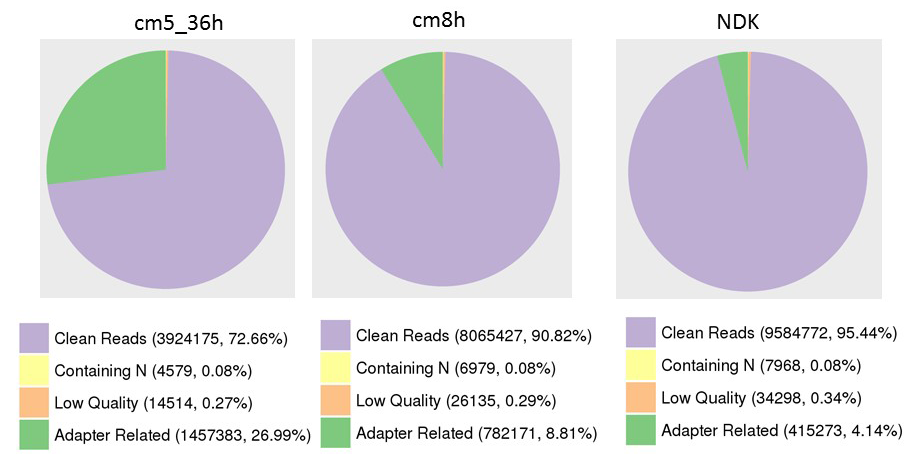

Supplement: Supplementary file 2 — Additional file 2: Fig. S2. Classification of Raw Reads of three samples (A) Classification of Raw Reads of Cm5_36h; (B) Classification of Raw Reads of Cm_8h; (C) Classification of Raw Reads of NDK. [file 12934_2019_1172_MOESM2_ESM.tif]

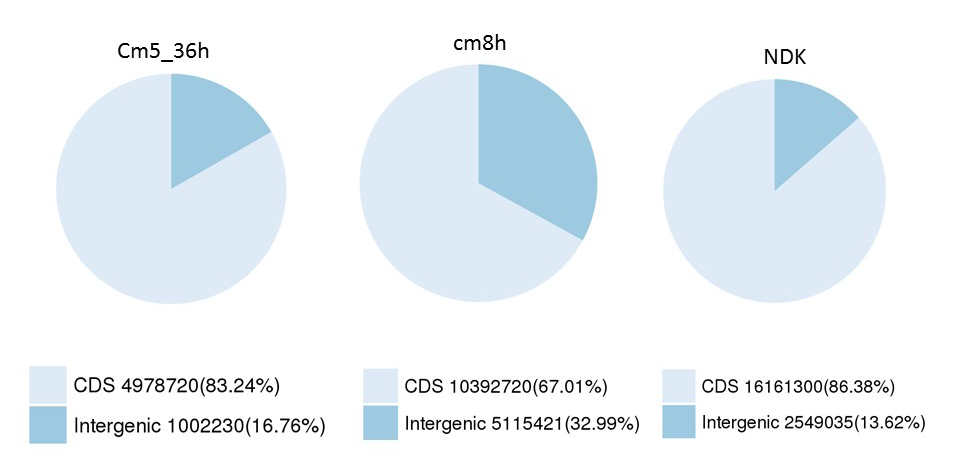

Supplement: Supplementary file 5 — Additional file 5: Fig. S3. Distribution of reads mapped to genomic regions of three samples. (A) Distribution of reads mapped to genomic regions in Cm5_36h; (B) Distribution of reads mapped to genomic regions in Cm_8h; (C) Distribution of reads mapped to genomic regions in NDK. [file 12934_2019_1172_MOESM5_ESM.tif]

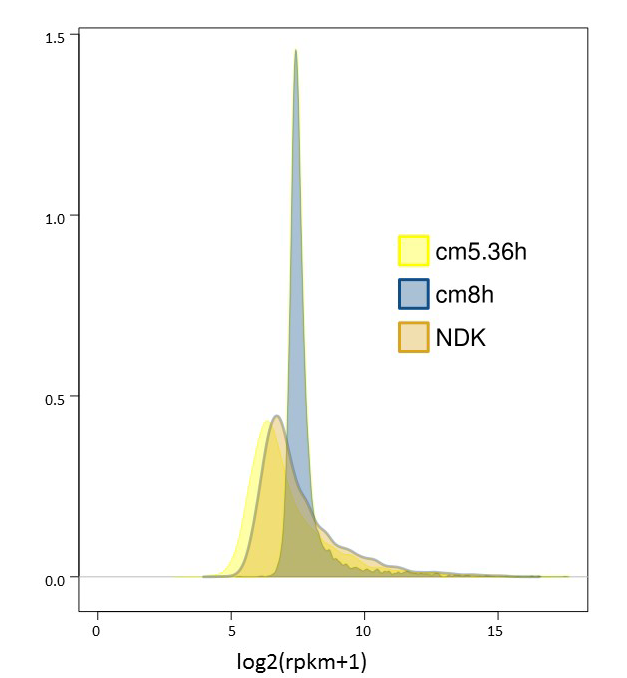

Supplement: Supplementary file 6 — Additional file 6: Fig. S4. RPKM distribution for all samples. [file 12934_2019_1172_MOESM6_ESM.tif]

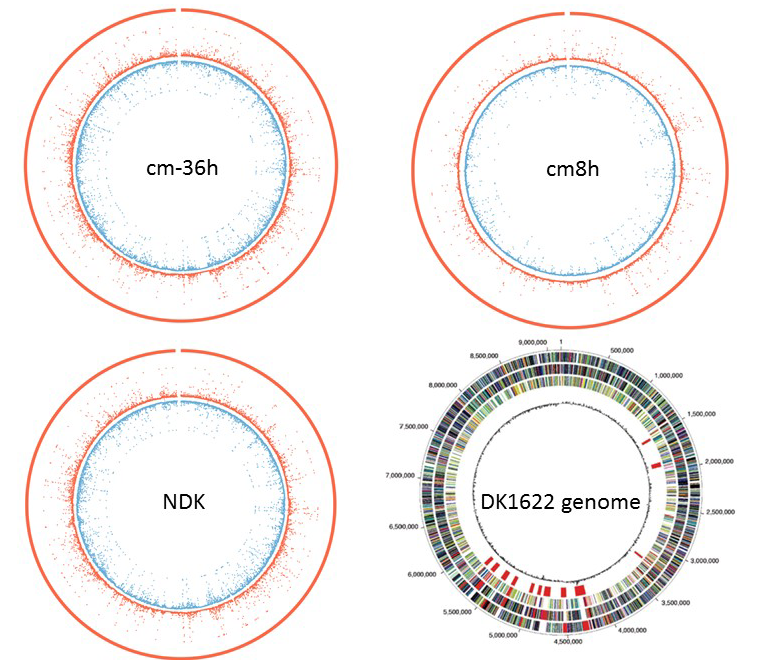

Supplement: Supplementary file 8 — Additional file 8: Fig. S5. Read density in the chromosomes of three samples (A) Read density of Cm5_36h in chromosomes; (B) Read density of Cm_8h in chromosomes; (C) Read density of NDK in chromosomes; (D) Genome map determined in Ref. [2]: layer 4 represents the biosynthetic gene cluster of secondary metabolites. [file 12934_2019_1172_MOESM8_ESM.tif]

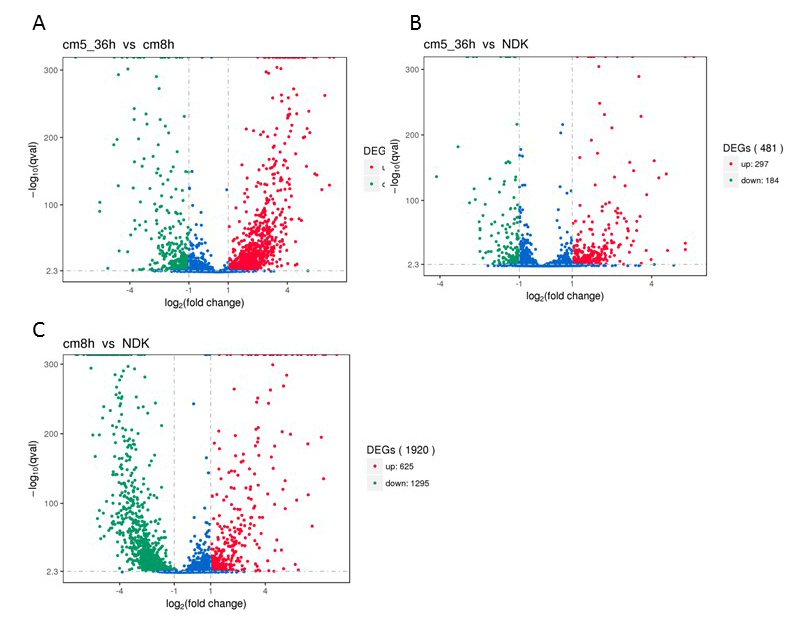

Supplement: Supplementary file 9 — Additional file 9: Fig. S6. The overall distribution of DEGs in three samples. [file 12934_2019_1172_MOESM9_ESM.tif]

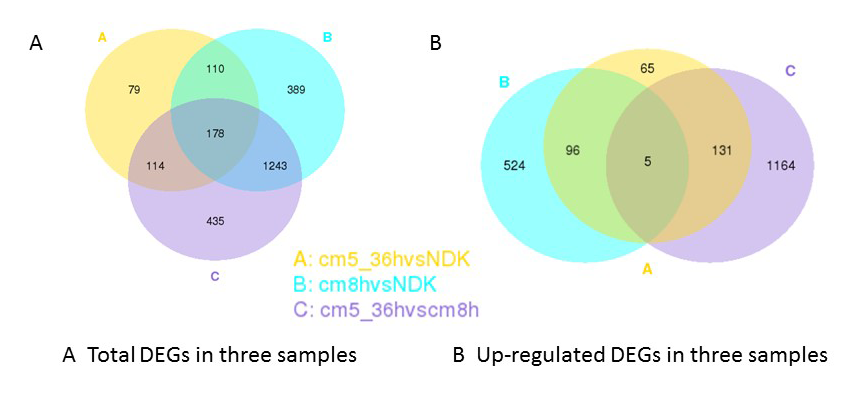

Supplement: Supplementary file 10 — Additional file 10: Fig. S7. Comparisons of the number and overlapping relationships of DEGs between different samples. A purple circle represents number of DEGs between Cm5_36h and Cm_8h; yellow circle stand for number of DEGs between Cm5_36h and NDK. The overlapping region means shared DEGs of two comparable groups. B. Purple circle represents number of DEGs between Cm5_36h and Cm_8h; yellow circle stand for number of DEGs between cm8h and NDK. The overlapping region means shared DEGs between two comparable groups. C. Purple circle represents number of DEGs between Cm_8h and NDK; yellow circle represents the number of DEGs between Cm5_36h and NDK. The overlapping region means shared DEGs between two comparable groups. [file 12934_2019_1172_MOESM10_ESM.tif]

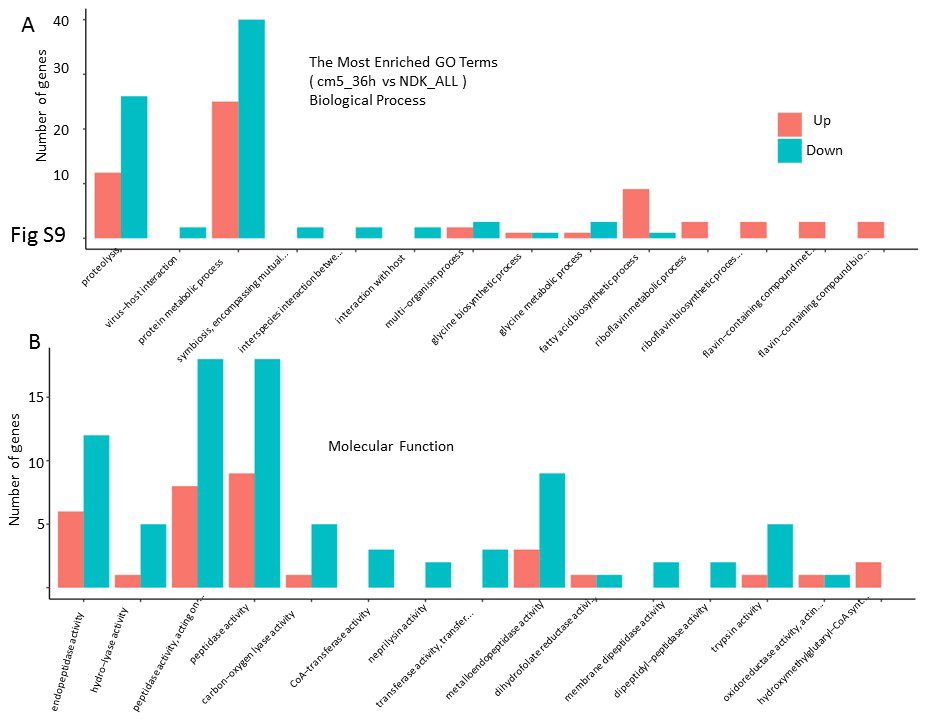

Supplement: Supplementary file 11 — Additional file 11: Fig. S8. The most enriched GO terms between Cm5_36h and NDK. [file 12934_2019_1172_MOESM11_ESM.tif]

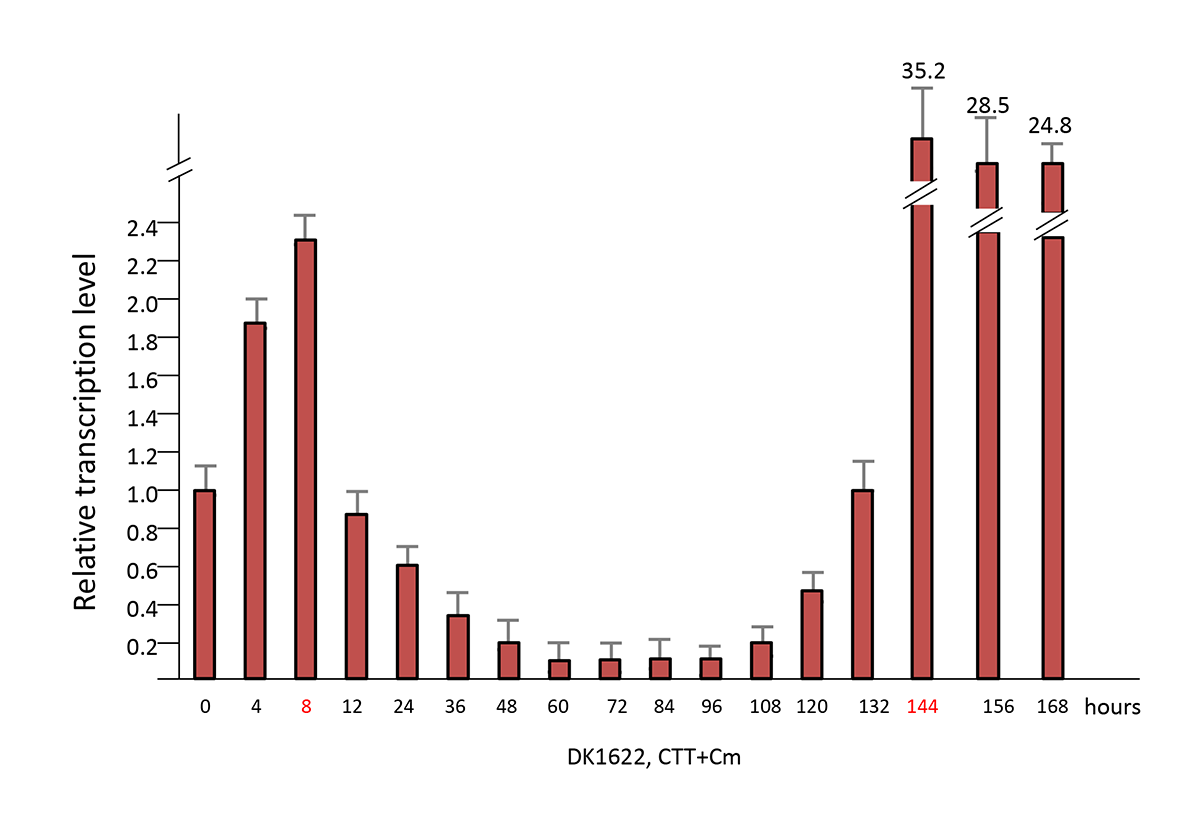

Supplement: Supplementary file 12 — Additional file 12: Fig. S9. The relative transcription level of MXAN_2566 of DK1622 in CTT + Cm by real time PCR using the same cell density. [file 12934_2019_1172_MOESM12_ESM.tif]
