# Supplementary material for: Whole transcriptome analysis and gene deletion to understand the chloramphenicol resistance mechanism and develop a screening method for homologous recombination in Myxococcus xanthus
Source: Microb Cell Fact. 2019 Jul 10;18:123. doi: 10.1186/s12934-019-1172-3 (PMC6617876; doi:10.1186/s12934-019-1172-3)
Supplement: Supplementary file 3 — Additional file 3: Table S1. Transcriptome data output of three samples. [file 12934_2019_1172_MOESM3_ESM.docx]

**Table S1** Transcriptome data output of three samples

| **Sample name** | **Raw reads** | **Raw bases** | **Clean reads** | **Clean bases** | | **Error rate** | **Q20** | **Q30** | **GC content** |
| --- | --- | --- | --- | --- | --- | --- | --- | --- | --- |
| Cm5_36h_1 | 5400651 | 0.81G | 3924175 | 0.59G | 0.01% | | 98.69% | 95.35% | 64.40% |
| Cm5_36h_2 | 5400651 | 0.81G | 3924175 | 0.59G | 0.01% | | 98.18% | 95.02% | 64.11% |
| Cm_8h_1 | 8880712 | 1.33G | 8065427 | 1.21G | 0.01% | | 98.69% | 95.29% | 67.67% |
| Cm_8h _2 | 8880712 | 1.33G | 8065427 | 1.21G | 0.01% | | 97.90% | 94.34% | 67.53% |
| NDK_1 | 10042311 | 1.5G | 9584772 | 1.44G | 0.01% | | 98.65% | 95.17% | 67.25% |
| NDK_2 | 10042311 | 1.5G | 9584772 | 1.44G | 0.01% | | 97.63% | 93.72% | 67.04% |
