# Supplementary material for: Whole transcriptome analysis and gene deletion to understand the chloramphenicol resistance mechanism and develop a screening method for homologous recombination in Myxococcus xanthus
Source: Microb Cell Fact. 2019 Jul 10;18:123. doi: 10.1186/s12934-019-1172-3 (PMC6617876; doi:10.1186/s12934-019-1172-3)
Supplement: Supplementary file 4 — Additional file 4: Table S2. Percentages of genes in different expression levels. [file 12934_2019_1172_MOESM4_ESM.docx]

**Table S2** Percentages of genes in different expression levels

| **FPKM Interval** | **cm5_36h** | **cm8h** | **NDK** |
| --- | --- | --- | --- |
| 0-1 | 43(0.59%) | 25(0.34%) | 45(0.61%) |
| 1-3 | 14(0.19%) | 13(0.18%) | 24(0.33%) |
| 3-15 | 199(2.71%) | 54(0.73%) | 278(3.78%) |
| 15-60 | 4537(61.73%) | 3166(43.07%) | 4758(64.73%) |
| >60 | 2557(34.79%) | 4092(55.67%) | 2245(30.54%) |
