# Supplementary material for: Whole transcriptome analysis and gene deletion to understand the chloramphenicol resistance mechanism and develop a screening method for homologous recombination in Myxococcus xanthus
Source: Microb Cell Fact. 2019 Jul 10;18:123. doi: 10.1186/s12934-019-1172-3 (PMC6617876; doi:10.1186/s12934-019-1172-3)
Supplement: Supplementary file 7 — Additional file 7: Table S3. Percentages of reads mapping to the reference genome. [file 12934_2019_1172_MOESM7_ESM.docx]

**Table S3**  Percentages of reads mapping to the reference genome

| **Sample name** | **Cm5_36h** | **Cm_8h** | **NDK** |
| --- | --- | --- | --- |
| Total reads | 7848350 | 16130854 | 19169544 |
| Total mapped | 6536786 (83.29%) | 15866665 (98.36%) | 19037259 (99.31%) |
| Multiple mapped | 763087 (9.72%) | 820008 (5.08%) | 674722 (3.52%) |
| Uniquely mapped | 5773699 (73.57%) | 15046657 (93.28%) | 18362537 (95.79%) |
| Read-1 | 2880299 (36.7%) | 7524695 (46.65%) | 9176151 (47.87%) |
| Read-2 | 2893400 (36.87%) | 7521962 (46.63%) | 9186386 (47.92%) |
| Reads map to '+' | 2887227 (36.79%) | 7522901 (46.64%) | 9181463 (47.9%) |
| Reads map to '-' | 2886472 (36.78%) | 7523756 (46.64%) | 9181074 (47.89%) |
