# Supplementary material for: Whole transcriptome analysis and gene deletion to understand the chloramphenicol resistance mechanism and develop a screening method for homologous recombination in Myxococcus xanthus
Source: Microb Cell Fact. 2019 Jul 10;18:123. doi: 10.1186/s12934-019-1172-3 (PMC6617876; doi:10.1186/s12934-019-1172-3)
Supplement: Supplementary file 13 — Additional file 13: Table S4. Differentially expressed genes related with fatty acid process in Cm5_36h. [file 12934_2019_1172_MOESM13_ESM.docx]

**Table S4** differentially expressed genes related with fatty acid process in Cm5-36h

| Gene Name | Log2FC | function | position |
| --- | --- | --- | --- |
| MXAN_3462 | 1.30 | polyketide synthase | unidentified myxalamid |
| MXAN_3932 | 1.15 | polyketide synthase | TA cluster |
| MXAN_3933 | 1.33 | polyketide synthase | TA cluster |
| MXAN_3935 | 1.30 | non-ribosomal peptide synthetase | TA cluster |
| MXAN_3936 | 1.30 | polyketide synthase | TA cluster |
| MXAN_3938 | 1.46 | polyketide synthase | TA cluster |
| MXAN_3941 | 1.32 | polyketide synthase | TA cluster |
| MXAN_4301 | 1.37 | polyketide synthase | DKxanthene |
| MXAN_6401 | 1.11 | beta-ketoacyl-[acyl-carrier-protein] synthase family protein | lantibiotics |
