# Supplementary material for: Whole transcriptome analysis and gene deletion to understand the chloramphenicol resistance mechanism and develop a screening method for homologous recombination in Myxococcus xanthus
Source: Microb Cell Fact. 2019 Jul 10;18:123. doi: 10.1186/s12934-019-1172-3 (PMC6617876; doi:10.1186/s12934-019-1172-3)
Supplement: Supplementary file 14 — Additional file 14: Table S5. Differentially expressed genes related with type 2 lantibiotic biosynthetic gene clusters in Cm5-36h. [file 12934_2019_1172_MOESM14_ESM.docx]

**Table S5** differentially expressed genes related with type 2 lantibiotic biosynthetic gene cluster in Cm5-36h

| Gene name | Log2FC | funtion |
| --- | --- | --- |
| MXAN_6389 | 1.47 | mersacidin/lichenicidin family type 2 lantibiotic |
| MXAN_6392 | 3.79 | acyl carrier protein |
| MXAN_6394 | 1.88 | MBL fold hydrolase |
| MXAN_6401 | 1.11 | beta-ketoacyl-[acyl-carrier-protein] synthase family protein |
| MXAN_6403 | 1.08 | ABC transporter permease |
| MXAN_6406 | 1.74 | hypothetical protein |
| MXAN_6407 | 1.06 | hypothetical protein |
