# Supplementary material for: Whole transcriptome analysis and gene deletion to understand the chloramphenicol resistance mechanism and develop a screening method for homologous recombination in Myxococcus xanthus
Source: Microb Cell Fact. 2019 Jul 10;18:123. doi: 10.1186/s12934-019-1172-3 (PMC6617876; doi:10.1186/s12934-019-1172-3)
Supplement: Supplementary file 15 — Additional file 15: Table S6. Differentially expressed genes related with protein biosynthesis in Cm5_36h. [file 12934_2019_1172_MOESM15_ESM.docx]

**Table S6** differentially expressed genes related with protein biosynthesis in Cm5-36h

| Gene name | Log2FC | funtion |
| --- | --- | --- |
| MXAN_1491 | 5.28 | tRNA-Tyr |
| MXAN_0380 | 5.27 | tRNA-Gly |
| MXAN_0388 | 4.60 | tRNA-Arg |
| MXAN_0381 | 2.94 | tRNA-Leu |
| MXAN_4702 | 3.98 | 23S ribosomal RNA |
