# Supplementary material for: Whole transcriptome analysis and gene deletion to understand the chloramphenicol resistance mechanism and develop a screening method for homologous recombination in Myxococcus xanthus
Source: Microb Cell Fact. 2019 Jul 10;18:123. doi: 10.1186/s12934-019-1172-3 (PMC6617876; doi:10.1186/s12934-019-1172-3)
Supplement: Supplementary file 16 — Additional file 16: Table S7. Primers used in this study. [file 12934_2019_1172_MOESM16_ESM.docx]

**Table S7** primers used in this study.

| Name | Sequence(5’-3’) |
| --- | --- |
| DRT-356F | CGGAATTCGGGGAGGCTGAGGAAGAGGATG |
| DRT-1280R | GCTCTAGACGGCATCGTGTTCTCCGTCTCC |
| DRT-1904F | GCTCTAGAGAGCCGAAGGCCGACGAACATC |
| DRT-2980R | CCAAGCTTCCCAACCACGGCGAATAGAACC |
| DRT-1169F(P21) | GCCGACGATGAGGCTCTGGATG |
| DRT-2247R  (P24) | CGGATGCAATCGTGCCTGACTT |
| 307f (P1) | GAATTCTCTAGAAAGCTTCCCAGCCCGCCTAATGAGC |
| 693r (P2) | TGTCAGCCGTTAAGTGTTCCTGTGTC |
| 719f | GACGTTGATCGGCACGTAAGAGG |
| 1534r | AATCTGACCTCCTGGTTATG |
| XE307F | AATCTAGAGAATTCCCCAGCCCGCCTAATGAGC |
| H-1534R | CCAAGCTTAATCTGACCTCCTGGTTATG |
| S11D- 141f | GCTCTAGAGCCCACTGAAGGCATCACGGTAAC |
| S11D-2093R | CCAAGCTTGCCGCTCCTCGTGCTTGAAGTAGA |
| S14D-1057f | GCTCTAGAACCGACCTGCTGAAATCAAACCTG |
| S14D-2649R | CCAAGCTTGTAGGAGGGTAAGGTGGAACGGAACT |
| P6-615R | TTGTCCACAACCGTTAAACC |
| P5-903F | AGTCAGTTGCTCAATGTACC |
| P3 | GTTACCGTGATGCCTTCAGTGGGCTCTAGA |
| P4 | TCTACTTCAAGCACGAGGAGCGGCAAGCTT |
